# Genes and Gene Functions Associated with Morphological, Productive, Reproductive, and Carcass Quality Traits in Pigs: A Functional Bioinformatics Approach

**DOI:** 10.3390/cimb48020153

**Published:** 2026-01-30

**Authors:** Wilber Hernández-Montiel, Víctor M. Meza-Villalvazo, Dany A. Dzib-Cauich, Juan M. Zaldívar-Cruz, José Abad-Zavaleta, Nubia Noemi Cob-Calan, Nicolás Valenzuela-Jiménez, Roberto Zamora-Bustillos, Amada I. Osorio-Terán

**Affiliations:** 1 Instituto de Agroingeniería, Universidad del Papaloapan, Av. Ferrocarril s/n, Ciudad Universitaria, Campus Loma Bonita, Loma Bonita 68400, Mexico; nvalenzuela@unpa.edu.mx; 2 Instituto de Biotecnología Animal, Universidad del Papaloapan, Circuito Central No. 200, Parque Industrial, Tuxtepec 68301, Mexico; meza1077@hotmail.com (V.M.M.-V.); joseabadz@hotmail.com (J.A.-Z.); 3 Instituto Tecnológico Superior de Escárcega, Calle 85 Entre 10b s/n Colonia: Unidad Esfuerzo y Trabajo, Escárcega 24350, Mexico; danidzib@escarcega.tecnm.mx; 4 Colegio de Postgraduados, Campus Tabasco, Periférico Carlos A. Molina S/N Km. 3, Huimanguillo 86500, Mexico; zaldivar@colpos.mx; 5 Departamento de Posgrado e Investigación, Instituto Tecnológico Superior de Calkiní, Av. Ah Canul S/N por carretera Federal, Calkiní 24900, Mexico; ncalan@itescam.edu.mx; 6 TecNM/Instituto Tecnológico de Conkal, Av. Tecnológico S/N, Conkal 97345, Mexico; roberto.zb@conkal.tecnm.mx

**Keywords:** bioinformatics, gene ontology, selection, pig genetics, reproductive traits, carcass quality

## Abstract

Understanding the functional mechanisms of genes influencing economically important traits in the domestic pig is essential for optimizing marker-assisted selection (MAS). This study aimed to characterize the biological functions, molecular mechanisms, and metabolic pathways of genes associated with morphological, productive, reproductive, and carcass quality traits through a functional bioinformatics approach. Genes were compiled from 116 peer-reviewed studies published between 2000 and 2024, and subsequently grouped according to trait. A de novo functional bioinformatics analysis was performed on this dataset. Functional enrichment analysis was conducted using DAVID and the clusterProfiler package in R, applying FDR correction (≤0.05). Protein-protein interaction (PPI) networks were explored using STRING. No individual gene was consistently reported with high frequency. Among the most frequently reported genes were *VRTN* (17 studies) for teat number, *HOMER1* (3 studies) for leg strength, and *BMPR1B* (3 studies) for litter size. Enriched GO terms included processes such as positive regulation of transcription (GO:0045944), chondrocyte differentiation (GO:0032331), and SMAD signaling (GO:0060391; an FDR = 7.34 × 10^−7^). The PPI networks revealed key genes involved in signaling and immune regulation. In conclusion, this bioinformatics analysis provides an integrated functional overview of the genes underlying key economic traits in pigs, identifying pleiotropic pathways such as SMAD/TGF-β signaling, which supports the development of more effective MAS strategies in pig breeding programs.

## 1. Introduction

Domestic pigs (*Sus scrofa* *f.* domestica) are among the most important livestock species worldwide due to their high productive and reproductive potential, providing approximately 35% of the total meat consumed globally [1]. For decades, genetic improvement programs have focused on identifying animals with superior production performance, aiming to optimize intensive production systems, particularly growth rate and feed conversion efficiency [2]. Genetic selection programs have traditionally prioritized traits such as weight gain, feed conversion ratio, and number of piglets born alive [3,4]. However, other traits of great economic and biological importance, such as body morphology, leg strength, teat number, and carcass quality are regulated by complex polygenic and multilevel genetic mechanisms. Therefore, elucidating the interactions and combined effects of these genes is essential to effectively integrate them into modern genetic selection and breeding schemes [5].

Advances in omics technologies and bioinformatics tools have enabled the identification of a large number of genes and single nucleotide polymorphisms (SNPs) associated with morphological, productive, reproductive, and meat quality traits in pigs [6,7]. However, the functional understanding of these genes remains limited. It is therefore essential to further elucidate their biological functions, mechanisms of action, and involvement in specific metabolic pathways [8]. Functional integration of these data is crucial for the efficient application of genetic knowledge in marker-assisted selection (MAS) programs [9].

Although pig breeding programs are primarily aimed at improving polygenic traits of economic importance, the final phenotypic expression is modulated by non-genetic factors, such as environmental conditions [10], and breed effects [11]. This interaction highlights the need to unravel the underlying genetic mechanisms governing these traits. Therefore, the identification of candidate genes and key biological pathways is a fundamental step toward optimizing breeding programs and enhancing the application of MAS and genomic selection strategies.

Functional annotation of livestock genomes is essential to understand the molecular mechanisms underlying complex traits of economic importance and contributes to the study of adaptive evolution and comparative genomics among species [12,13]. Gene Ontology (GO) resource serves as a central framework for functional genomics research; GO annotations are widely used for hypothesis generation and are often combined with high-throughput biological data, improving the interpretation of genetic and genomic results [14]. In parallel, PPI networks are valuable tools for systematically investigating complex biological activities within cells [15].

Functional annotation thus provides a more comprehensive understanding of the roles of genes in animal phenotypes and establishes the foundation for MAS-based selection strategies with improved efficiency, targeting traits of economic and zootechnical value, and offering potential applications in genome editing of pig breeds.

This study therefore employed a functional bioinformatics approach to characterize the biological roles, molecular mechanisms, and associated metabolic pathways of genes influencing key morphological, productive, reproductive, and carcass quality traits in pigs. To achieve this, bioinformatics tools such as DAVID and clusterProfiler were employed to compare and interpret gene functions.

## 2. Materials and Methods

### 2.1. Database

This study conducted a functional bioinformatics analysis on the gene dataset previously compiled and reported by Dzib-Cauich et al. [5], which includes a total of 116 open-access scientific articles. Of these, 20 were excluded for not reporting specific genes associated with the target traits. Consequently, 96 articles met the inclusion criteria and were used for gene extraction. The complete list of the 96 studies included, along with the genes extracted from each one categorized by trait, is provided in Supplementary Table S1. The search for these articles was performed in the PubMed, Mendeley, ScienceDirect, Springer, and Google Scholar databases, covering studies published between 2000 and 2024, following a systematic synthesis approach [16]. The extracted information was organized using LibreOffice Calc (v5.0), including the following parameters: author, year, title, journal, abstract, issue, volume, pages, breed, traits (productive, reproductive, and carcass), genes, and molecular tool/model.

### 2.2. Gene Selection and Inclusion/Exclusion Criteria

The genes selected for analysis were grouped according to trait category (Table S1). These genes were annotated from the studies analyzed in Dzib-Cauich et al. [5]. All genes reported in the scientific literature between 2002 and 2024 that were associated with the traits of interest: morphology, limb strength, teat number, litter uniformity, prolificacy, and carcass and meat quality in domestic pigs (*Sus scrofa f*. domestica) were included.

Only studies reporting molecular associations (e.g., PCR, SNPs, QTL, GWAS, differential expression, or functional validation assays) and providing the name of the gene(s) were considered for inclusion. Studies reporting gene-trait associations without functional validation were not excluded, provided they reported a specific gene name, as the primary goal was to compile a comprehensive gene list for subsequent bioinformatic analysis.

### 2.3. Gene Frequency, Functional Enrichment (GO), and Metabolic Pathways

The frequency of gene occurrence in the published studies was calculated using the tidyverse package (v2.0.0) [17], in R (v4.4.1). An initial functional enrichment analysis (GO) and metabolic pathway identification (KEGG Pathway) were performed using DAVID Bioinformatics Resources v2024q4 (https://davidbioinformatics.nih.gov/home.jsp (accessed on 7 July 2025)).

Subsequently, a second analysis was conducted using the clusterProfiler package (v4.12.0) [18] in R, applying the org.Ss.eg.db annotation database (v3.19.1) for *Sus scrofa f.* domestica. To ensure consistency, we used the official gene identifier (Entrez Gene ID or standard gene symbol) for each reported gene, without distinguishing among specific isoforms. Functional analyses (GO, KEGG) were based on the canonical gene annotation, assuming that the main functions are conserved across isoforms. Semantic networks of GO terms were generated using the GOSemSim package (v2.32.0) in R, which computes semantic similarity between GO terms based on their ontological structure and shared gene annotations [19].

Enrichment plots (dotplots) were generated using tidyverse (v2.0.0) and ggplot2 (v4.0.1) in R, displaying the 20 most significant GO terms (lowest FDR) for each trait. The selection for presentation was thus based solely on statistical significance. Subsequently, the biological interpretation and discussion of these terms focused on their relevance to the studied porcine traits. The significance of the enrichment was determined using a False Discovery Rate (FDR) correction, with a threshold of FDR ≤ 0.05 considered statistically significant.

### 2.4. Protein-Protein Interaction (PPI) Networks (STRING)

Functional interactions among the proteins encoded by the analyzed genes were explored using STRING v12.0 (https://string-db.org/ (accessed on 22 August 2025)). For each trait, the PPI network was built using all genes involved in significantly enriched GO terms (FDR ≤ 0.05).

Only interactions with a confidence score threshold ≥ 0.7 (high confidence) were considered, as this is a standard and recommended cutoff in the STRING database to ensure high reliability of the predicted interactions while minimizing false positives [20].

The analysis incorporated known and predicted interactions based on experimental evidence, curated databases, and co-expression data, with database access on 31 July 2024. Hub genes within each network were identified quantitatively based on their connectivity, specifically using the ‘degree’ centrality metric (the number of direct interactions a node has). Genes with a degree value in the top 10% of their respective network were defined as hubs for subsequent analysis.

Networks were visualized to highlight key nodes (hub genes) and functional clusters, using the protein annotation and functional annotation output files generated by STRING.

### 2.5. Code Availability

The R scripts used for gene frequency calculation, functional enrichment analysis with clusterProfiler, and generation of plots are available in a public GitHub repository (https://github.com/wilbermontiel11-droid/R-scripts-porcine (accessed on 2 January 2026)).

## 3. Results

### 3.1. Gene Frequency, GO Enrichment Analysis, and Semantic Network

**Figure 1:**
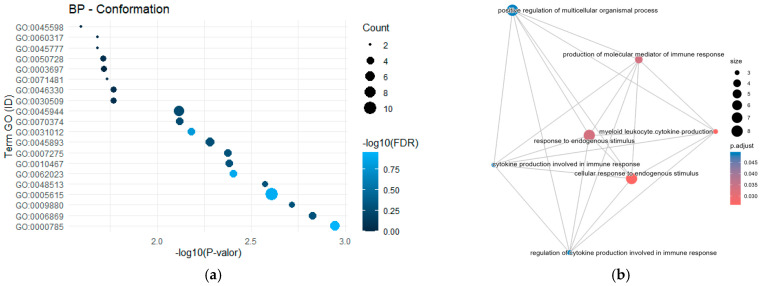
Functional enrichment analysis of candidate genes for body conformation. (**a**) Significantly enriched GO Biological Process terms; (**b**) Network visualization showing the relationships between enriched terms, highlighting central processes like muscle organ development and regulation of multicellular organismal processes.

No single gene stood out with consistently high frequency across all traits; however, several genes were recurrently reported for specific traits, indicating trait-dependent genetic associations. For the morphological traits GO analysis for Biological Processes revealed the term GO:0045944-positive regulation of transcription by RNA polymerase II, enriched with seven genes (*NFYA*, *IHH*, *NUCKS1*, *BMPR1B*, *TLR4*, *WNT2* and *PPARD*) and an FDR value of 0.571 (Figure 1a). Although this term did not reach statistical significance, its identification suggests a biological trend that could be further explored in studies with greater statistical power.

It is important to clarify that the frequency of a gene’s occurrence across the compiled studies primarily reflects the degree of research attention it has received in the literature for a given trait. This metric is useful for identifying recurrent candidate genes but does not directly correlate with their effect size, functional impact, or causal relationship to the phenotype. This interpretive caveat applies to all gene frequency mentions throughout the results section.

The semantic network visualization (Figure 1b) illustrates the functional interrelationships among the enriched GO terms, showing a clustering pattern primarily associated with immune and cell signaling functions, suggesting potential shared regulatory pathways among genes linked to body conformation. The most significant term was “*positive regulation of multicellular organismal process*,” which displayed high connectivity with other biologically related terms.

**Figure 2:**
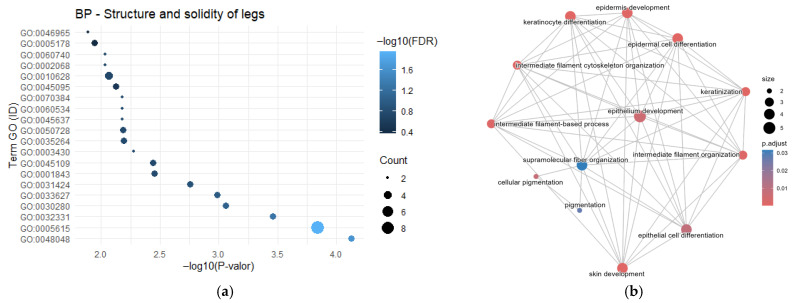
Gene Ontology analysis reveals processes underlying genetic variants for leg strength. (**a**) Enriched GO Biological Process terms highlighting the role of striated muscle development and aerobic respiration; (**b**) Semantic similarity network where cluster colors group terms into cohesive functional modules, emphasizing the theme of muscle system process.

Genes associated with leg strength in pigs have been previously reported. In this study, the *GLP1R* gene was identified in two independent studies, whereas *HOMER1* appeared in three studies. The GO enrichment analysis revealed 20 annotated classifications (Figure 2a). The term GO:0010628-positive regulation of gene expression was enriched with four genes (*RARG*, *CALCR*, *ITGB7* and *WNT16*), while GO:0032331-negative regulation of chondrocyte differentiation was enriched with three genes (*RARG*, *IHH* and *ITGB7*), showing an FDR of 0.053. Conversely, the semantic network (Figure 2b) displayed multiple interactions among the terms “*epithelial cell differentiation*”, “*skin development*”, and “*keratinocyte differentiation*”, indicating a high level of significance and suggesting shared molecular mechanisms related to tissue differentiation and limb development.

**Figure 3:**
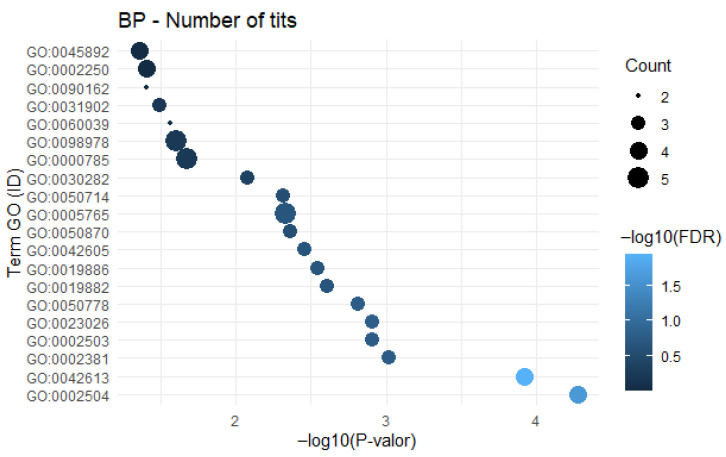
Functional enrichment of candidate genes for porcine teat number. Bar chart showing the top 10 significantly enriched GO Biological Process terms (FDR < 0.05).

For teat number, the *VRTN* (Vertebrae Development Associated) gene was identified with a frequency of 17 studies, followed by *ABCD4*, reported in four studies, and *PTHLH* (Parathyroid Hormone-Like Hormone), reported in two studies. These results indicate that *VRTN*, *ABCD4* and *PTHLH* exhibit recurrent frequency across studies, suggesting a strong association with body conformation traits. A total of 71 genes were included in the GO analysis performed on both platforms. The GO enrichment analysis revealed 22 annotated biological process classifications (Figure 3). The terms GO:0002504-antigen processing and presentation of peptide or polysaccharide antigen via MHC class II, and GO:0002250-adaptive immune response, were enriched with four genes (*SLA-DQB1*, *SLA-DRB1*, *SLA-DQA1* and *SLA-DRA*), showing FDR values of 0.024 and 0.993, respectively.

The term GO:0050714-positive regulation of protein secretion showed suggestive enrichment (*p*-value = 0.0049), involving three genes (*TGFB3*, *FRMD4A* and *ARF6*). However, this result did not survive strict multiple-testing correction (FDR = 0.27) and is therefore not statistically significant according to our threshold. It is presented here as a suggestive biological trend that merits validation in future, targeted studies. No semantic network was plotted for the genes reported in relation to teat number.

For the litter uniformity trait, only the genes *SMAD2* and *BMP2* were identified, each reported in two independent studies. A total of 140 genes were included in the Gene Ontology analysis using the DAVID platform. The GO enrichment analysis for Biological Process (BP) terms revealed a significant enrichment for GO:0008283-cell population proliferation, which involved five genes (*SMAD2*, *NDST1*, *SMAD4*, *PDGFA* and *IGF1*) with a false discovery rate (FDR) of 0.012034.

**Figure 4:**
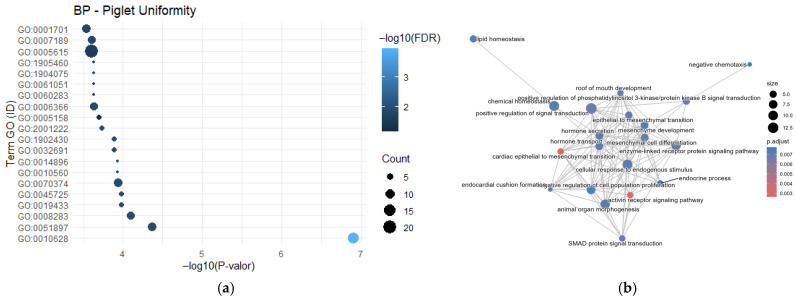
Functional profiling of genes associated with litter uniformity. (**a**) Significantly enriched GO Biological Processes highlight pathways involved in prenatal development and endocrinology; and (**b**) Network visualization groups these processes into functional modules, illustrating the complex interplay of systems underlying this reproductive trait.

Additionally, the term GO:0010628-positive regulation of gene expression was found to be highly enriched, comprising twelve genes (*SMAD2*, *SEC16B*, *SMAD4*, *BMP2*, *CALCR*, *IL1B2*, *GPER1*, *KPNA7*, *INHBA*, *IGF1*, *WNT16* and *APOB*) with an FDR of 1.17 × 10^−4^ (Figure 4a).

The semantic network (Figure 4b) of genes associated with litter uniformity revealed multiple interactions among the *activin receptor signaling pathway* and *cellular response to estradiol stimulus*, as well as additional processes including *hormone secretion*, *hormone transport*, *SMAD protein signal transduction*, and *lipid homeostasis*.

**Figure 5:**
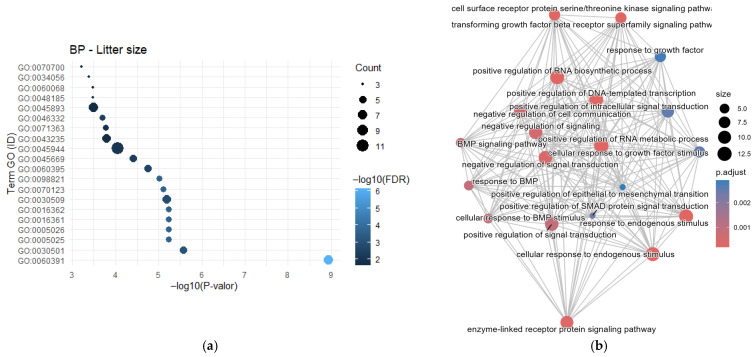
Functional characterization of candidate genes for litter size. (**a**) Significant enrichment of GO Biological Processes related to embryonic development, uterine receptivity, and blood vessel formation; and (**b**) Network visualization groups these terms into interconnected functional modules, illustrating the multifaceted biological basis of this key reproductive trait.

For the litter size (prolificacy) trait, four genes (*ESRRG*, *MAP1LC3A*, *ESR1* and *ZFYVE9*) were identified with a frequency of two studies each, while the gene *BMPR1B* was reported in three studies. A total of 75 genes were annotated and included in the GO analysis using the DAVID platform (Figure 5a).

The GO term GO:0060391~positive regulation of SMAD protein signal transduction was enriched in seven genes (*ACVR1*, *INHBA*, *BMP7*, *ACVR2A*, *BMP6*, *BMP5*, and *TGFBR2*), showing an FDR of 7.34 × 10^−7^. The semantic network (Figure 5b) reveals a distribution of functional and biological links related to reproductive activity, highlighting the TGF-β/BMP signaling pathway, which is mediated by serine/threonine kinase receptors, as well as SMAD proteins and the intracellular signaling cascade. A general activation of gene transcription processes, such as “*RNA biosynthetic process*” and “*DNA-templated transcription*”, was also observed. These processes suggest a role in maintaining epithelial homeostasis and in the cellular response to stimuli associated with reproductive activity in sows.

**Figure 6:**
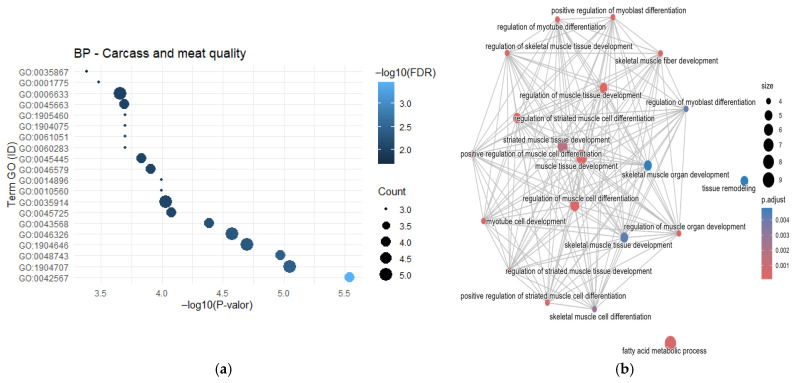
Functional profiling of genes influencing carcass composition and meat quality. (**a**) Significantly enriched GO Biological Processes highlight pathways central to myogenesis, adipogenesis, and extracellular matrix organization; and (**b**) network visualization groups these terms into functional modules, illustrating the biological complexity underlying these economically crucial traits.

For carcass quality traits, only two studies were found reporting the frequency of each of the following genes: *ACACA*, *GRM4*, *TNNI1* and *TNNI2*. A total of 119 genes were annotated in the GO analysis. Among these, the most significant genes identified using DAVID were *IGF1*, *MYOG*, *MYOD1*, *MYF6*, *MYF5*, *FOS* and *IGFBP*, whereas in clusterProfiler, the most relevant genes were *MYOG*, *MYF5*, *MYOD1*, *MYF6*, *BMP2*, *IGFBP5*, *AKIRIN2*, *SGCZ* and *METTL8*. The GO terms associated with these traits include GO:0045663-positive regulation of myoblast differentiation (IGF1), GO:0035914-skeletal muscle cell differentiation (*MYOG*, *MYOD1*, *MYF6*, *FOS* and *MYF5*), GO:1904707~positive regulation of vascular-associated smooth muscle cell proliferation (*ERN1*, *IGFBP5* and *IGF1*), and GO:0042567~insulin-like growth factor ternary complex (*IGFBP5* and *IGF1*) (Figure 6a).

The semantic network for genes reported in association with carcass and meat quality traits revealed strong interactions among the terms “*regulation of muscle organ development*”, “*skeletal muscle tissue development*” and “*regulation of myoblast differentiation*”. This indicates a close relationship between muscle tissue development and differentiation and fatty acid metabolism, suggesting that these genes influence key meat quality attributes such as tenderness, marbling, texture, and consistency (Figure 6b).

### 3.2. Protein–Protein Interaction Networks (STRING)

**Figure 7:**
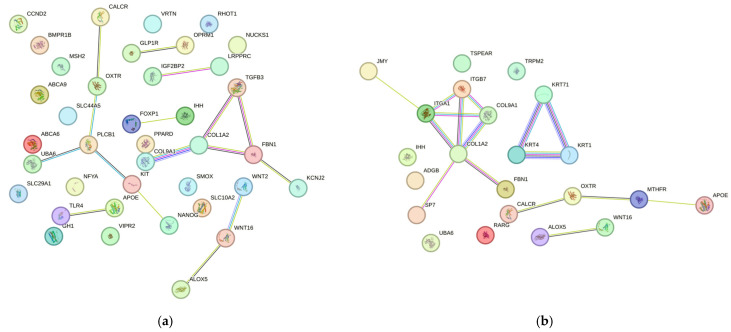
STRING network of genes associated with the traits: (**a**) conformation, and (**b**) structure and leg strength. The thickness of the edges indicates the strength of the interaction.

To understand the functional relationships among the genes, a PPI analysis was performed using STRING. For the conformation trait, the PPI network highlighted hub genes including *COL1A2*, *TGFB3*, and *FBN1*, with the *COL1A2-FBN1* interaction showing the highest confidence score (0.867). Across all traits, notable interactions included: *SMAD4-SMAD2* (0.999) for litter uniformity, *ACVR2A-INHBA* (0.987) for litter size, *HLA-DRA-SLA-DRB1* (0.936) for teat number, and *ACACA-FASN* (0.999) for meat quality (Table S2). The analysis revealed genes with high connectivity (hub genes, high node degree), highlighting proteins such as *COL1A2*, *TGFB3*, *FBN1*, *WNT2*, *WNT16* and *KIT*, which showed the greatest number of interactions within the network (Figure 7a). The *OPRM1* gene was identified in the metabolic pathways *ssc04081: Hormone signaling* and *ssc04080: Neuroactive ligand–receptor interaction*.

For the structure and leg strength trait, the PPI analysis included 21 related genes (Figure 7b). The network contained 15 key genes: *JMY*, *TSPEAR*, *ITGB7*, *TRPM2*, *KRT71*, *COL9A1*, *KRT4*, *KRT1*, *FBN1*, *CALCR*, *RARG*, *ALOX5*, *WNT16*, *MTHFR* and *APOE*. The proteins showing the highest interaction were enriched in the metabolic pathways *ssc04512: ECM–receptor interaction* and *ssc04151: PI3K–Akt signaling pathway*, particularly involving the genes *COL1A2*, *ITGB7*, *COL9A1* and *ITGA1*.

**Figure 8:**
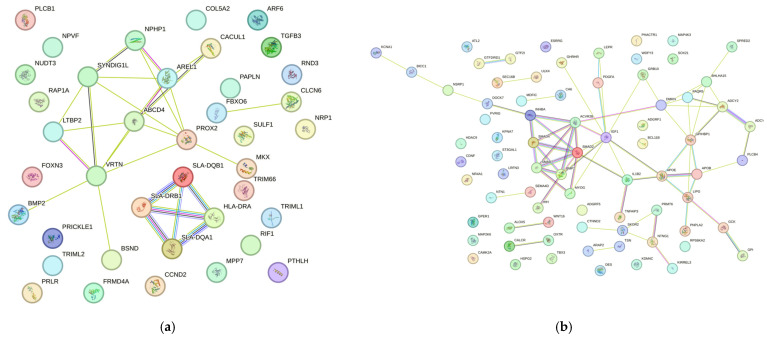
STRING network of genes associated with the traits: (**a**) number of teats and (**b**) litter uniformity.

The PPI network analysis for the number of teats, an important trait related to milk production, included a total of 41 genes analyzed using STRING. The generated network revealed several highly connected nodes (hubs), with *ABCD4*, *AREL1*, *PROX2* and *VRTN* showing the highest number of interactions within the network (Figure 8a). This suggests a possible functional coordination among these genes in determining the number of teats. The metabolic pathway with a potential effect on mammary gland development was *ssc05321: Inflammatory bowel disease*, identified in the *TGFB3* gene. The PPI network analysis for the litter uniformity trait, which reflects the even growth of piglets within a litter, included a total of 76 genes analyzed using STRING. Among the most highly connected hubs were *ACVR2B*, *BMP2*, *SMAD2*, *SMAD4*, *SMAD7*, *IGF1*, *DMKN* and *BHLHA15* (Figure 8b). The enriched signaling pathways associated with these genes included ssc04550: Signaling pathways regulating pluripotency of stem cells, *ssc04350: TGF-beta signaling pathway*, *ssc04935: Growth hormone synthesis*, *secretion and action*, and *ssc04913: Ovarian steroidogenesis*.

**Figure 9:**
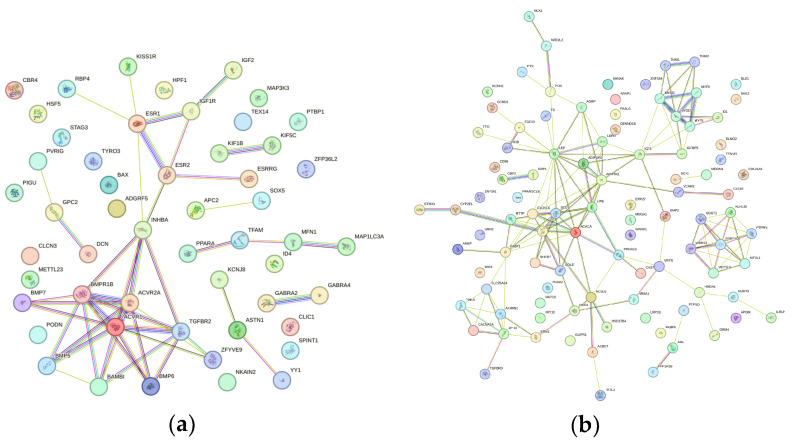
STRING network of genes associated with the traits: (**a**) litter size and (**b**) carcass and meat quality.

The PPI network analysis for the litter size trait, which reflects the overall growth performance of the litter, included a total of 52 genes analyzed using STRING. The analysis revealed a set of central hub nodes primarily involved in the TGF-β and BMP signaling pathways, which play essential roles in embryonic development and cell proliferation. Among the identified hub genes were *ACVR1*, *ACVR2A*, *BMPR1B*, *BMP5*, *BMP6*, *BMP7*, *INHBA*, *TGFBR2*, *ESR1* and *ESR2* (Figure 9a). In addition, peripheral nodes such as *ASTN1*, *GPC2*, *PVRIG*, *KIF1B* and *KIF5C* were observed, exhibiting fewer interactions and potentially acting as modulators of specific biological processes in pigs.

The PPI network analysis for the carcass and meat quality trait, an economically important characteristic, revealed regulation primarily through metabolic processes. A total of 99 genes were analyzed using STRING. Among the central hub genes identified were *ACACA*, *FASN*, *SCD*, *ELOVL6*, *ADIPOQ*, *LEP* and *PRKAG3* (Figure 9b). The main metabolic pathways associated with carcass and meat quality included *ssc04152: AMPK signaling pathway*, *ssc04920: Adipocytokine signaling pathway*, *and ssc01212: Fatty acid metabolism*.

## 4. Discussion

This study provides an integrated overview of the functional mechanisms underlying morphological, productive, reproductive, and carcass and meat quality traits in pigs.

For carcass quality traits, the GO terms associated with these traits include GO:0045663-positive regulation of myoblast differentiation (IGF1) and GO:0035914-skeletal muscle cell differentiation.

### 4.1. Morphological Characteristics

Morphometric traits are recognized as key phenotypic indicators in livestock and play a crucial role in phenotype-based selection strategies [21]. The functional enrichment analysis performed using DAVID identified *FBN1*, *COL1A2*, *PLCB1*, *KIT*, *WNT16* and *APOE* as the principal genes associated with phenotypic conformation. Furthermore, the STRING network analysis revealed additional enrichment of *APOE*, *VIPR2*, *CALCR* and *GLP1R*, supporting their involvement in extracellular matrix organization and bone development. These findings suggest that the coordinated activity of these genes contributes to the structural integrity and morphogenetic architecture that underlie body conformation in pigs.

It has been observed that the *FBN1* (Fibrillin-1) gene contributes to both the structural and instructive information that flows to and from the cell, orchestrating tissue formation, homeostasis, and repair [22]. The *FBN1* gene is strongly linked to *TGF-β3* (Figure 7a) within conformation-related traits, as one of the principal functions of *FBN1* is its ability to regulate the bioavailability and activation of *TGF-β3*, a key growth factor involved in tissue and skeletal system development. The regulation and association between *FBN1* and *TGF-β3* are of critical importance in the organism, since it is well known that mutations in the *FBN1* gene can cause an autosomal dominant disease that affects connective tissue. This condition is characterized by various abnormalities in the bones, ocular tissues, and the cardiovascular system [23]. Moreover, a strong expression and correlation were also observed with the *COL1A2* (Collagen Type I Alpha 2 Chain) gene, which encodes type I collagen. Variants of this gene have been associated with low bone mass and increased susceptibility to fractures [24].

On the other hand, the STRING analysis revealed a strong relationship among *APOE*, *UBA6* and *PLCB1*. The *APOE* (Apolipoprotein E) gene is a component of plasma lipoproteins that plays an important role in the transport and metabolism of cholesterol and other lipids [25]. The *UBA6* (Ubiquitin Like Modifier Activating Enzyme 6) gene encodes an activating enzyme that regulates numerous cellular processes, including protein turnover, iron homeostasis, and cell development [26]. Meanwhile, *PLCB1* (Phospholipase C Beta 1) encodes a protein that enables the use of calcium as a cofactor and plays a key role in the intracellular transduction of multiple extracellular signals. The results obtained in the present study confirm the findings reported by Fan et al. [27], reported that the *CALCR* and *COL1A2* genes are significantly associated (*p* < 0.05) with calcium metabolism and body size traits (length, depth, and width). Overall, these associations provide evidence of the strong interrelationship among genes and proteins involved in lipid and protein metabolism, as well as calcium regulation processes that are fundamental to conformation-related traits.

Similarly, according to the results obtained from the GO and STRING analyses, the conformation trait is polygenic and does not depend on a single gene. Although gene expression is important, the genotype-environment interaction is responsible for determining the phenotype. Furthermore, gene expression depends on allelic frequencies as well as on transcriptional regulatory patterns that are characteristic of each population [28].

For the leg strength trait, the main genes identified were *RARG*, *ITGB7*, *FBN1*, *KRT4*, *COL1A2*, *IHH* and *KRT71*, as determined by the functional analysis performed using DAVID (Figure 2a). The *RARG* gene (Retinoic Acid Receptor Gamma) encodes a retinoic acid receptor that functions as a potent cellular regulator and belongs to the nuclear hormone receptor family. Liu et al. [29], demonstrated that inhibition of *RARG* in mesenchymal cells increases the expression of chondrogenic genes such as *Sox9* and *Col2a1*, suggesting a direct regulatory role in the cartilage formation pathway. Moreover, mice with the Rarg^Δ/Δ genotype exhibit reduced trabecular bone mass and an increased number of trabecular osteoclasts. Therefore, *RARG* expression in Prrx1-Cre–targeted cells directly regulates endochondral bone formation and indirectly influences tibial vascularization [30].

The STRING analysis for the leg strength trait (Figure 7b) revealed a strong association among different gene clusters. On one side, a strong interaction was observed among *ITGB7*, *ITGB1*, *COL9A1*, *COL1A2* and *SBN*; on the other side, a marked association was identified among *KRT71*, *KRT1* and *KRT4*.

The *ITGB7* (Integrin Subunit Beta 7) gene encodes a protein belonging to the integrin superfamily. This gene is regulated by kisspeptin, which acts by increasing progesterone secretion and promoting cell proliferation, thereby reducing apoptosis in ovarian granulosa cells [31]. As previously mentioned, the *FBN1* (Fibrillin 1) gene is an essential component of the extracellular matrix microfibrils. In skeletal tissues, despite its low abundance, *FBN1* is fundamental for the organization of elastic and collagen fibers and for regulating the bioavailability of growth factors such as TGF-β and BMP [32]. Fibrillin-rich microfibrils are present in all connective tissues and form part of the structural framework specific to each tissue, conferring essential physical and mechanical properties-either alone or together with elastin within elastic fibers [33]. In humans, mutations in the *FBN1* gene have been identified with negative effects on the structure and function of the limbs [34]. Consistent with the associations observed in this study by Fan et al. [27], reported that genes such as *APOE*, *CALCR*, *OXTR*, *OPG* and *MTHFR* directly influence leg strength and locomotion. In the present analysis, the COL1A2 gene stands out, as it appears in two main network nodes by Body Conformation and Leg Strength, highlighting its biological relevance (Figure 8b).

On the other hand, the *KRT71*, *KRT4* and *KRT1* genes are involved in the formation of keratin filaments and other structural proteins essential for the assembly of keratin networks [35]. Keratin is a key protein responsible for the structural integrity of skin, hair, and nails; therefore, the interactions observed among these genes indicate that they are crucial elements for the selection of productive traits in pigs.

Finally, the STRING network analysis for the Leg Strength trait revealed a strong association between *WNT16* and *ALOX5*. The *WNT16* (Wnt Family Member 16) gene plays a fundamental role in the establishment of digit patterning during limb development in vertebrates. Meyers et al. [36] reported that *WNT16* acts as a combined mitogenic and pro-osteogenic stimulus, potentially playing a functional role in bone repair mediated by human mesenchymal stem cells. This gene is therefore significantly involved in osteogenic differentiation during developmental stages. In contrast, the *ALOX5* (Arachidonate 5-Lipoxygenase) gene is implicated in the regulation and proliferation of myoblasts, playing an essential role in muscle growth and development [37]. Consequently, mutations or overexpression of this gene could directly affect muscle formation and overall growth performance.

Considering the results from the GO and PPI analyses for the conformation trait, the genes analyzed demonstrated their importance through their ability to regulate both morphological integrity and locomotor functionality.

### 4.2. Productive Performance

Productive performance is one of the most important parameters within production systems. These traits are largely determined by the females, in which the phenotype–environment interaction influences piglet weight during growth and at weaning [38,39].

The genes identified for number of teats, enriched in the DAVID analysis, included *BMP2*, *TGFB3*, *PRLR*, *PTHLH*, *PRICKLE1*, *PROX2* and *MKX*. In contrast, for milk production, the enriched genes were *PRLR*, *TGFB3*, *BMP2*, *PTHLH*, *SLA-DQA1*, *SLA-DRB1*, *SLA-DQB1* and *CCND2*.

The *BMP2* (Bone Morphogenetic Protein 2) gene plays a key role in chondrogenesis, a process involved in the formation of cartilage from mesenchymal cells, which is crucial for teat development. Wang et al. [40], reported that a combination of *BMP2* and *TGF-β3* promotes the chondrogenic differentiation of bone marrow-derived mesenchymal stem cells (BMSCs). In Duroc × (Landrace × Yorkshire) crossbred pigs, a mutation (rs320706814) in the *BMP2* gene affects carcass length due to its role in bone growth and development [41]. Hong et al. [42], reported an association between the *BMP2* and *ABCD4* genes and their influence on body length in sows, which consequently affects the number of teats. The correlation between these genes suggests that a greater body length in sows corresponds to a higher number of teats available for piglets. Indeed, in Duroc × (Landrace × Yorkshire) hybrid pigs, among the genes associated with body weight and conformation, *BMP2* was identified as a pleiotropic gene due to its effects on multiple traits in these hybrids [43]. Meanwhile, the *TGFB3* (Transforming Growth Factor Beta 3) gene is involved in several important cellular functions, including cell proliferation and differentiation, as well as the growth and development of theca and granulosa cells [44]. On the other hand, the *PRLR* (Prolactin Receptor) gene has been shown to influence growth traits in livestock [45]. In Holstein cattle, two polymorphisms (g.9206G > A and g.9681C > T) have been identified, both exhibiting a positive effect on milk production [46]. In indigenous Hungarian pigs, the most advantageous AA genotype was the least frequent (8.75%), whereas the BA and BB genotypes accounted for 40% and 51.25%, respectively. However, the AA genotype was associated with the largest litter size [47].

On the other hand, the STRING analysis for the number of teats trait revealed a strong interaction among *ABCD4*, *AREL*, *VRTN*, *LTBP2*, *NPHP1*, *CACUL1* and *SYNDIG1L*. Likewise, a strong association was also observed among *SLA-DRB1*, *SLA-DQB1*, *SLA-DQA1* and *SLA-DRA* (Figure 9a).

The strong correlation among *SLA-DRB1*, *SLA-DQB1*, *SLA-DQA1* and *SLA-DRA* can be explained by the fact that these genes belong to the porcine leukocyte antigen (SLA) class II complex, which comprises highly polymorphic gene clusters that play an active role in immune response. These genes encode cell-surface glycoproteins involved in responses to vaccines, infectious diseases, and productive traits. It has been reported that pigs carrying homozygous SLA haplotypes at immunologically relevant loci exhibit greater consistency in their immune responses to allografts and xenografts [48], suggesting that the expression of this gene complex could serve as a useful selection criterion for animals intended for xenotransplantation studies.

For Litter uniformity (birth and weaning weight of piglets), the genes showing the highest significance in the DAVID analysis were *IGF1*, *BMP2*, *SMAD2/4*, *INHBA*, *PDGFA*, *GHRHR*, *CALCR*, *GPER1*, *APOE* and *APOB*. In contrast, the most significant genes identified using clusterProfiler were *SMAD4*, *BMP2*, *TBX3*, *WNT16*, *SMAD7*, *INHBA*, *ACVR2B*, *GPER1* and *IGF1*.

The *IGF1* (Insulin-Like Growth Factor 1) gene regulates growth factors and energy metabolism in domestic animals [49]. In lambs from different breeds and their crosses, a SNP (g.0.195C > T) in the IGF1-R gene has been identified, showing a significant effect on growth traits [50]. In Landrace and Lantang pigs, the hepatic mRNA and/or serum protein levels of members of the IGF system are significantly correlated at different ages, acting in coordination to regulate growth performance and carcass composition in these breeds [51]. A study by Pierzchała et al. [52], reported breed-specific differences in the hepatic gene expression of *GHR*, *IGF1R*, *IGF1* and *IGF2* at various developmental stages in primiparous gilts, with the highest expression level of *IGF1* observed in Pietrain gilts at 60 and 90 days of age. The *SMAD2/4/7* (SMAD Family Member 2, SMAD Family Member 4, and SMAD Family Member 7) genes are components of the Transforming Growth Factor Beta (TGF-β) signaling pathway and exhibit transcriptional activity. Their phosphorylation activity has been observed in association with myostatin, promoting muscle fiber growth [53]. However, there is limited information available regarding the role of *SMAD* genes in developmental processes.

Moreover, the STRING analysis for the litter uniformity trait revealed strong associations among *BMP2*, *SMAD2*, *SMAD7*, *SMAD4*, *INHBA*, *ACVR2B* and *MYOG*, as well as a significant interaction between *ADCY2* and *ADCY*. The close relationship between *BMP2* and *SMAD* genes can be explained by their joint participation in the TGF-β signaling pathway, which regulates cellular activity and tissue growth. Similarly, the strong correlation between *ADCY* and *ADCY2*, illustrated in Figure 9b, reflects the influence of multiple genes on litter uniformity. Wang et al. [54] reported that *ADCY* and *ADCY2* are involved in key processes related to embryonic development, energy metabolism, and cell differentiation, underscoring their functional role in determining this trait.

Taken together, these findings emphasize the importance of considering the interaction among multiple genes and signaling pathways to better understand the genetic variability of reproductive traits in pigs, providing molecular evidence supporting the link between gene expression and reproductive phenotype.

### 4.3. Reproductive

Reproductive activity in both females and males is regulated by multiple genes; however, heritability is generally low. Consequently, reproductive failure is a common reason for the culling of females from production systems [55].

Genes associated with reproduction. Among the genes showing the highest significance for litter size are *ACVR1*, *ACVR2A*, *BMPR1B*, *BMP7*, *BMP6*, *BMP5*, *INHBA* and *TGFBR2*. In contrast, the genes most significantly enriched in the clusterProfiler analysis were *SMAD2*, *SMAD3*, *SMAD4*, *GDF9*, *BMPR2*, *FSHR*, *ESR1* and *LHCGR*.

The *ACVR1* (Activin A Receptor Type I) and *ACVR2A* (Activin A Receptor Type IIA) genes belong to the TGF-β ligand family, which performs multiple biological functions in embryonic stem cells as well as in differentiated tissues [56]. These genes are also crucial for skeletal development, including the formation of bones and cartilage [57]. A study conducted by Li et al. [58], in Yorkshire pigs with high and low prolificacy identified the SNP rs342908929, located near the ACVR1 gene, showing a significant association (*p* < 0.05) with total number born (TNB), number born alive (NBA), and litter weight in this breed. The *SMAD2/3/4* (SMAD Family Member 2, SMAD Family Member 3, and SMAD Family Member 4) genes play a major role in ovarian function, particularly in follicular development, atresia, and follicle selection [59,60]. In Tibetan sheep, two loci have been identified: g.10729C > T in the *SMAD1* gene (genotype CC) and g.21447C > T in the *SMAD3* gene (genotype TT), both of which are associated with increased litter size (*p* < 0.05 [59]. The *ESR1* (Estrogen Receptor 1) gene in females is also associated with increased litter size [61,62].

The STRING analysis confirmed the GO analysis for the litter size trait, highlighting a strong interaction among *INHBA*, *BMP7*, *BMPR1B*, *BMP5*, *BMP6*, *BAMBI*, *ACVR*, *ACVR2A* and *TGFBR2* (Figure 9a). Bone morphogenetic proteins (BMPs) belong to the transforming growth factor (TGF)-β superfamily, a group of signaling molecules that mediate a wide range of biological processes, from early embryonic tissue formation to postnatal tissue homeostasis [63]; Therefore, the strong relationships observed in the STRING network underscore the importance of these genes’ expression in genetic selection for this trait.

Additionally, the STRING analysis revealed a significant association between the *ESR1*, *ESR2*, *IGF1R*, *IGF2* and *ESRRG* genes and litter size. The estrogen receptors (*ESR1* and *ESR2*) function as transcription factors predominantly expressed in female reproductive tissues, where they play a crucial role in regulating hormonal processes. According to Muñoz et al. [62], the *ESR2* gene participates in estrogenic functions related to follicular maturation and embryonic development during the peri-implantation phase. Similarly, *IGF2* is involved in signaling mechanisms associated with cell growth and differentiation. Taken together, the differential expression and close relationship of these genes with fertility suggest their determinant contribution to the genetic variability of litter size in pigs.

Other significant associations were identified for the *KIF1B*, *KIF5C*, *GABRA2* and *GABRA4* genes (Figure 9a). These results are consistent with those reported by Zhang et al. [64], who indicated that these and other related genes are potential candidates that provide new insights into the genetic basis of litter traits in pigs, which could be useful for improving reproductive performance in sow breeding programs.

The *KIF1B* (Kinesin Family Member 1B) and *KIF5C* (Kinesin Family Member 5C) genes belong to the kinesin family, which encodes motor proteins involved in intracellular transport along microtubules. In pigs, their expression has been associated with key cellular processes in reproduction, including oocyte maturation, apoptosis, and cell communication [65]. Genetic variations within these loci may alter the efficiency of these mechanisms, thereby influencing fertility and embryonic survival, which in turn directly affects litter size.

### 4.4. Carcass and Meat Quality

The most significant genes associated with carcass and meat quality identified through the DAVID analysis were *MYOG*, *MYOD1*, *MYF5*, *MYF6*, *FASN*, *SCD*, *LEP*, *ADIPOQ*, *BMP2* and *IGF1*, while those showing the highest significance in the clusterProfiler analysis included *FOS*, *IGFBP5*, *TGFBR3*, *FGF23*, *LEPR*, *ELOVL6*, *PPARGC1B*, *VCAM1*, *PTN* and *CCND2*.

The *MYOD1* (Myogenic Differentiation 1) gene belongs to the myogenic regulatory factor (MRF) family [66] and plays a key role in myogenesis and, consequently, in determining muscle fiber characteristics [67]. The *MYOG*, *MYOD1* and *MYF5* genes function in a regulatory cascade, controlling the expression of myogenic regulatory factors within different prenatal and postnatal myogenic cells [68]. In Yorkshire and Berkshire pig breeds, two SNPs g.489C > T and g.1264C > A, were identified in the *MYOD1* gene; these polymorphisms are significantly associated with muscle fiber traits, loin eye area, and meat lightness [69]. The *FASN* (Fatty Acid Synthase) gene encodes an enzyme responsible for the de novo synthesis of long-chain fatty acids. During oncogenesis, it plays a role in cell growth and survival, rather than functioning solely in energy storage pathways [70]. A study by Dzib-Cauich et al. [71], evaluated the expression of genes related to fatty acid profiles and lipid metabolism (*SCD*, *SREBP1*, *FASN* and *ACP*) in Pelón Mexicano and Landrace-Yorkshire pig breeds. The results indicated that these genes have a significant effect in the Pelón Mexicano breed, where their expression contributes to a higher intramuscular fat content in the *Longissimus dorsi* muscle.

The *SCD* (Stearoyl-CoA Desaturase) gene plays a key role in intramuscular fat content and is responsible for the biosynthesis of oleic acid (C18:1) from stearic acid (18:0) [72]. A study conducted by Estany et al. (2014), in Duroc pigs and their crossbreeds provided evidence of three SNPs (g.2108C > T; g.2228T > C; g.2281A > G) located in the promoter region, which were associated with an increased 18:1/18:0 ratio in both muscle and subcutaneous fat, but not in the liver [73]. In this regard, Liu et al. [74], demonstrated that low expression of the *SCD* gene impairs porcine adipocyte differentiation. The *LEPR* (Leptin Receptor) gene, located on SSC6, influences backfat thickness (BF), fat area proportions, and serum leptin concentration (LEPC) [75]. Moreover, *LEPR* is associated with fatty acid composition in muscle tissue and contributes to increased intramuscular fat, resulting in a higher proportion of saturated fatty acids (SFA), lower levels of polyunsaturated fatty acids (PUFA), and an increased SFA/PUFA ratio [72]. In Hanwoo cattle, two loci (g.16024A > G and g.10329C > T) have been identified as being associated with fatty acid composition, adipogenic gene expression, and carcass traits, revealing a systemic interaction between genetic factors and adipogenesis in this breed [76].

The STRING analysis revealed a strong interaction among the genes *MYF6*, *MYOG* and *MYOD1; EPHX1*, *CYP2E1*, *MTTP*, *FASN* and *ACACA*; as well as *WDR33*, *NF3L1*, *UGGT1*, *PRPF40A* and *METTL8*, all associated with meat and carcass quality traits (Figure 9b).

A study conducted by Cho et al. [77] on the functional relevance of the *EPHX1* gene demonstrated that the nsSNP c.685T > G showed significant effects on meat color, protein content, collagen content, and in 24 h. These findings suggest that the c.685T > G polymorphism in *EPHX1* may serve as a promising DNA marker for improving economically important traits in pigs.

Ropka-Molik et al. [78], reported that the *MTTP* gene can be considered a candidate gene influencing pork meat quality traits. The expression of *MTTP* (Microsomal Triglyceride Transfer Protein) is involved in lipid transport between membrane vesicles, a key mechanism for the assembly of chylomicrons, low-density lipoproteins (LDL), and very-low-density lipoproteins (VLDL). The biological importance of this protein lies in the fact that, in both humans and pigs, mutations in the *MTTP* locus have been shown to affect lipid transfer activity.

The genes *FASN* and *ACACA* exhibited a strong association with meat quality traits. These results are consistent with those reported by Piórkowska et al. [79] who evaluated mutations in the *SCD*, *ACACA* and *FASN* genes in relation to pork meat quality and other production traits. Their findings indicated that selection for the *FASN* A allele in Polish Large White pigs could improve meat quality characteristics such as water-holding capacity and meat color.

## 5. Conclusions

In conclusion, this integrative study demonstrates that economically important traits in pigs are regulated by complex and interconnected genetic networks. The identification of high-frequency genes such as *VRTN* and *BMPR1B*, together with the consistent enrichment in specific biological pathways such as SMAD/TGF-β and BMP signaling, underscores a shared molecular basis (pleiotropy) among morphological, reproductive, and carcass quality traits. The PPI network analysis confirmed the centrality of these genes within critical functional modules. Altogether, this functional characterization provides a valuable resource for genomic selection, enabling the prioritization of candidate genes and pleiotropic pathways for the simultaneous and more efficient improvement of multiple traits in pig breeding programs.

## Data Availability

The raw data supporting the conclusions of this article will be made available by the authors on request.

## Supplementary Materials

The following supporting information can be downloaded at: https://www.mdpi.com/article/10.3390/cimb48020153/s1. References [27,42,43,54,58,62,64,80,81,82,83,84,85,86,87,88,89,90,91,92,93,94,95,96,97,98,99,100,101,102,103,104,105,106,107,108,109,110,111,112,113,114,115,116,117,118,119,120,121,122,123,124,125,126,127,128,129,130,131,132,133,134,135,136,137,138,139,140,141,142,143,144,145,146,147,148,149,150,151,152,153,154,155,156,157,158,159,160,161,162,163,164,165,166,167,168] are cited in the Supplementary Materials.
