# Supplementary material for: Genes and Gene Functions Associated with Morphological, Productive, Reproductive, and Carcass Quality Traits in Pigs: A Functional Bioinformatics Approach
Source: Curr Issues Mol Biol. 2026 Jan 30;48(2):153. doi: 10.3390/cimb48020153 (PMC12939253; doi:10.3390/cimb48020153)
Supplement: Supplementary file 1 [file cimb-48-00153-s001.zip › Table S1. Genes and reference.pdf]

Tabla S1. Genes reported and their references, associated with morphological characteristics, productive and reproductive behavior, and carcass and meat quality in pig breeds.

Trait	Count	Genes	Reference
Conformation	12	*OPRM1, SLC44A5, WASHC4, NOPCHAP1, RHOT1,* ***GLP1R****, TGFB3, PLCB1, TLR4, KCNJ2, ABCA6, ABCA9*	Gao et al. [80]
	7	*VIPR2, SLC10A2, NUCKS1, MCT1, CHCHD3, SMOX, GPR1*	Liu et al. [81]
	9	***GLP1R****, NFYA, NANOG, COX7A2, BMPR1B, FOXP1, SLC29A1, CNTNAP4, KIT*	Lan et at. [82]
	8	*LRPPRC, WRAP73, VRTN, PPARD, IGF2BP2, GH1, CCND2, MSH2*	Le et al. [83]
	6	*MTHFR, WNT2, APOE, BMP8, GNRHR, OXTR*	Fan et al. [84]
	15	*APOE, BMP8, CALCR, COL1A2, COL9A1, DKFZ, FBN1, VDBP, ALOX5, OPG, OXTR, WNT16, GNRHR, IHH, MTHFR*	Fan et al. [27]
Structure and Solidity of the legs	3	***HOMER1****, JMY, ITGA1, RAB32*	Hao et al. [85]
	1	***HOMER1***	Schumacher et al. [86]
	1	***HOMER1***	Xu et al. [87]
	11	*KRT71, KRT1, KRT4, ITGB7, CSAD, RARG, SP7, PFKL, TRPM2, SUMO3, TSPEAR*	Zhou et al. [88]
	18	*APOE, BMP8, CALCR, COL1A2, COL9A1, DKFZ, FBN1, VDBP, ALOX5, BMP8, CALCR, OPG, OXTR, WNT16, GNRHR, IHH, MTHFR, WNT16*	Fan et al. [27]
Number of tets	6	***VRTN****, Prox2, MPP7, ARMC4, MKX, δ-EF1*	Duijvesteijn et al. [89]
	6	*BMP2,* ***ABCD4****,* ***VRTN****, AREL1, PLCB1, CACUL1*	Hong et al. [42]
	1	***VRTN***	Lopes et al. [90]
	1	***VRTN***	Rohrer and Nonneman [91]
	7	*HEG1, XYLT1, SULF1, MUC13,* ***VRTN****, RAP1A, NPVF*	Wei et al. [92]
	9	*ANO3, MUC15, DISP3, FBXO6, CLCN6, HLA-DRA, SLA-DRB1, SLA-DQB1, SLA-DQA1*	Shi et al. [93]
	5	*NUDT3,* ***VRTN****, COL5A2, BSND, CARTPT*	Bian et al. [94]
	1	*TBC1D21*	Jin et al. [95]
	7	*PTGR2, FAM161B, LIN52,* ***VRTN****, FCF1, AREL1, LRRC74A*	Tan et al. [96]
	5	*TRIML1, TRIML2, ZFP42, FAT1, MTNR1A*	Liu et al. [97]
	1	***VRTN***	Uzzaman et al. [98]
	7	*PTP4A2, NPHP1, CYP24A1, YLPM1, SYNDIG1L, TGFB3,* ***VRTN***	Verardo et al. [99]
	1	***VRTN***	Zhuang et al. [100]

	6	*SPATA6*, ***VRTN***, *FOXN3*, *KCNK10*, *RND3*, *RIF1*	Chen et al. [101]
	1	***VRTN***	Yang et al. [102]
	2	*PRLR*, ***VRTN***	Hong et al. [103]
	4	***VRTN***, *SYNDIG1L*, *DPF3*, *NRP1*	Li et al. [104]
	2	*TRIM66*, *PRICKLE1*	Lin et al. [105]
	1	***PTHLH***	Martínez-Giner et al. [106]
	5	*MC4R*, *CCND2*, ***VRTN*** *ABCD4*, *BMP2*	Nosková et al. [107]
	8	***VRTN***, *JAZF*, *LTBP2 FRMD4A*, *ARF6*, *PAPLN*, *KATNIP*, *AREL1*	Park et al. [108]
	2	***PTHLH***, *PTHR1*	Tetzlaff et al. [109]
	2	***VRTN***, ***ABCD4***	van Son et al. [110]
	1	*LEF-1*	Xu te al. [111]
	1	***ABCD4***	Yang et al. [112]
	2	***ABCD4***, ***VRTN***	Yang et al. [113]
	9	*LOC100738627*, *SMAD7*, *LIPG*, *ACAA2*, *GTF2IRD1*, *GTF2I*, *LOC102157744*, *LOC102162346*, *UBTFL1*	Wang et al. [114]
	15	*CCDC3*, *LRRC8C*, *LRFN3*, *TNFRSF19*, *ESRRG*, *GHRHR*, *TUSC3*, *NBAS*, *BCL11B*, *CDNF*, *ULK4*, *CC2D2A*, *KCNK2*, *CCDC3*, *MINDY4*	Bakoev et al. [115]
	11	*APOE*, *CALCR*, *COL1A2*, *GNRHR*, *IHH*, *MTHFR*, *WNT16*, *ALOX5*, *BMP8*, *OPG*, *OXTR*	Fan et al. [27]
	2	*NR4A1*, *GNB2L1*	Kumchoo and Mekchay [116]
	5	*SMAD4*, *RPS6KA2*, *CAMK2A*, *NDST1*, *ADCY5*	Sun et al. [117]
Pigler uniformity	14	*BHLHA15*, *OCM2*, *IL1B2*, *GCK*, ***SMAD2***, *HABP2*, *PAQR5*, *GRB10*, *PRELID2*, *DMKN*, *GPI*, *GPIHBP1*, *ADCY2*, *ACVR2B*	Wang et al. [54]
	11	*TMEM184B*, *TNFRSF9*, *CA6*, *CCDC37*, *TBC1D19*, *ADH4*, *WDFY3*, *LOC100738652*, *HDAC9*, *MDFIC*, *BCOR*	Zhang et al. [118]
	14	*TXN2*, *KCNA1*, *ENSSSCG00000003546*, *ZDHHC18*, *MAP2K6*, *BICC1*, *FAM135B*, *EPHB2*, *SEMA4D*, *ST3GAL1*, *KCTD3*, *FAM110A*, *TMEM132D*, *TBX3*	Zhang et al. [119]
	8	*INHBA*, *LEPR*, *HDHD2*, *CTNND2*, *RNF216*, *HMX1*, *PAPPA2*, *NTN1*	Zhao et al. [120]
	5	*ax7*, *Myf5*, *MyoD*, *DES*, *MyoG*	Stange et al. [121]
	2	*ADGRF1*, *ADGRF5*	Sell-Kubiak et al. [122]
	4	*AACS*, *APOB*, *OSBPL10*, *LRP1B*	Wang et al. [123]

	11	*INTS10, KIRREL3, SOX21,* ***BMP2****, MAP4K3, SOD3, FAM160B1, ATL2, SPRED2, SEC16B, RASAL2*	Deng et al. [124]
	7	*TSN, NIFK, CLASP1, ARAP2, PHACTR1, PVRIG, ZCWPW1*	Lee et al. [125]
	7	*SKOR2,* ***SMAD2****, VAV3, NTNG1, SLC25A24, PRMT6, STXBP3*	Li et al. [58]
	4	*PDGFA, GPER1, PNPLA2, BSCL2*	Qiu et al. [126]
	2	*NSRP1, DOCK7*	Wu et al. [127]
	6	*TNFAIP3, KDM4C, HSPG2,* ***BMP2****, PLCB4, GRM5*	Zhang et al. [43]
	2	*IGF1, ESR*	Korwin-Kossakowska et al. [128]
	2	*MDR1, OPN*	Ni et al. [129]
	1	*GPR54*	Wu et al. [130]
	1	*KPNA7*	Zhang et al. [131]
Litter Size	9	***ESRRG****, MFN1, GPC2, STAG3, PVRIG, IRX1,* ***MAP1LC3A****, PIGU, ZFP36L2*	Cielén et al. [132]
	2	*NAT9, MAP3K3*	Hwang et al. [133]
	2	***ESR1****, ESR2*	Muñoz et a. [62]
	2	*IL-4, IL-4R*	Norseeda et al. [134]
	2	*BAX,* ***BMPR1B***	Sun et al. [135]
	1	*METTL23*	Sun et al. [136]
	6	*HMGB1, SOX5, KCNJ8, ABCC9, YY1, ASTN1*	Wu et al. [137]
	20	*NKAIN2, IGF1R, KISS1R, TYRO3, SPINT1, ADGRF5, APC2, PTBP1, CLCN3, CBR4, HPF1, FAM174A, SCP2, CLIC1,* ***ZFYVE9****, SPATA33, KIF5C, EPC2, GABRA2, GABRA4*	Zhang et al. [64]
	1	***BMPR1B***	Zhao et al. [138]
	1	***BMPR1B***	Liu et al. [139]
	3	*RBP4,* ***ESR1****, IGF2*	Muñoz et al. [140]
	1	*MMP-9*	Niu et al. [141]
	11	*BMP5, BMP6, BMP7, ACVR1, INHBA,* ***ZFYVE9****, TGFBR2, DCN, ID4, BAMBI, ACVR2A*	Li et al. [58]
	5	*PPARA, TFAM, ER1, ER2,* ***ESRRG***	Liu et al. [142]
	6	*TEX14, SEP4, HSF5, CYP26B1, SCD5, PCF11*	Zhang et al. [143]
calidad de la canal y carne	5	*DHCR7, FGF23, MEDAG, DGKI, PTN*	Gozalo-Marcilla et al. [144]
	1	*SQLE*	Ha et al. [145]
	1	*HSD17B4*	Jo et al. [146]

8	*PRKAG3, CAST, ADIPOQ, ELOVL6, FASN, MTTP,* ***ACACA****, NR6A1*	Palma-Granados et al. [147]
4	*TKTL2, ACBD7, ACSL6, FOS*	Wang et al. [148]
20	*TENM3, VRTN,* ***GRM4****, CCND2, BMP2, UNC5D, WARS1, MAP1S, ERP27, UBN2, SLC25A45, ATRNL1, LRP1B, KL, SGCZ, CD96, HAO1, RFTN1, NDUFS4, MINDY4*	Xie et al. [149]
3	*FKBP5, MDGA1, ILRUN*	Zong et al. [150]
3	*MC4R, LEP, H-FABP*	Chao et al. [151]
12	*PTPRD, PPARGC1B, VCAM1, SLC35A3, AGL, ADIPOR2, VDR, MYOG, FASLG, NCK1, CYP2E1, ID1*	Fernández et al. [152]
1	*DKK2*	He et al. [153]
2	*MYF5, MYOD1*	Liu et al. [154]
14	*LEPR,* ***ACACA****, FTO, LIPE, SCD, LRIG3, DENND1B, SOWAHB, EPHX1, NFE2L2, KRT10, NLE1, KCNH2, AHNAK*	Óvilo et al. [155]
1	*APOM*	Pan et al. [156]
1	*DIO3*	Qiao et al. [157]
2	*YOG, MYF6*	Wyszyňska-Koko et al. [158]
9	*LEKHB2, WDR33, NIF3L1, IGFBP5, UGGT1, PRPF40A, METTL8, PTPRN, KLHL30*	Daza et al. [159]
1	*GLIPR1*	Liu et al. [160]
2	*AMBP, PPP1R3B*	Cinar et al. [161]
4	*DOK7, ARAP1, ELMO2, SLC13A3*	Ding et al. [162]
11	*FABP1, AKIRIN2, GLP2R, TGFBR3, JPH3, ICAM2, ERN1, SLC25A14, IGF1, PI4KA, CACNA1A*	Jeong et al. [163]
2	***TNNI1, TNNI2***	Ngu et al. [164]
4	*GBP1, GBP2, CD163, CD169*	Niu et al. [165]
1	*c-MYC*	Oh et al. [166]
8	*COL21A1, ZNF184, ZNF391, HMGA1,* ***GRM4****, NUDT3, PGM2L1, PLBD2*	Wang et al. [167]
2	***TNNI1, TNNI2***	Yang et al. [168]

Note: All references cited in this table are included in the bibliography of the main article.
