# Supplementary material for: Genes and Gene Functions Associated with Morphological, Productive, Reproductive, and Carcass Quality Traits in Pigs: A Functional Bioinformatics Approach
Source: Curr Issues Mol Biol. 2026 Jan 30;48(2):153. doi: 10.3390/cimb48020153 (PMC12939253; doi:10.3390/cimb48020153)
Supplement: Supplementary file 1 [file cimb-48-00153-s001.zip › Table S2. Protein-protein interactions corresponding to high-, medium-, and low-degree nodes identified using the STRING database..pdf]

Table S2. Protein–protein interactions corresponding to high-, medium-, and low-degree nodes identified using the STRING database.

Interaction Level	Number of Nodes	Node 1	Node 2	Co-expression	Combined Score	Estimated Biological Relevance
**Conformtion trait**	**32**					
High		*COL1A2*	*FBN1*	0.659	0.867	Extracellular matrix structure.
High		*KIT*	*PLCB1*	0.067	0.659	Cell signaling and receptor tyrosine kinases.
Medium		*APOE*	*TLR4*	0.073	0.616	Relationship between lipid metabolism and inflammation.
Medium		*KIT*	*NANOG*	0	0.578	Maintenance of cellular pluripotency.
Low		*OPRM1*	*GLP1R*	0.051	0.502	Weak interaction between G protein-coupled receptors.
Low		*ALOX5*	*WNT16*	0.053	0.408	Functional association based primarily on text mining.
**leg strength trait**	**32**					
High		*COL1A2*	*FBN1*	0.867	0.659	Extracellular matrix and ligament stability.
High		*COL1A2*	*ITGA1*	0.803	0.049	Adhesion of bone/tendon cells to the extracellular matrix.
Medium		*COL1A2*	*ITGB7*	0.679	0.051	Cell-cell communication and structural support.
Medium		*ITGA1*	*ITGB7*	0.686	0	Formation of integrin complexes for signaling.
Medium		*COL1A2*	*COL9A1*	0.589	0	Organization of different collagen types within tissues.
Low		*KRT4*	*KRT1*	0.583	0.087	Integrity of epithelial tissues and hooves.
Low		*SP7*	*COL1A2*	0.463	0	Regulation of osteoblast differentiation.
Low		*ALOX5*	*WNT16*	0.408	0.053	Signaling pathways involved in bone density.
**number of teats trait**	54					
High		*SLA-DRA*	*SLA-DRB1*	0.936	0.443	Immune recognition complex and udder health.
High		*SLA-DQA1*	*SLA-DRB1*	0.904	0.299	Antigen presentation and resistance to mastitis.
High		*PROX2*	*VRTN*	0.871	0	*VRTN* is a key gene controlling vertebral and teat number.

High		*ABCD4*	*VRTN*	0.858	0	Regulation of metabolic transport during development.
Medium		*SYNDIG1L*	*VRTN*	0.847	0.073	Nervous system development and tissue organization.
Medium		*AREL1*	*PROX2*	0.775	0	Modulation of cellular signaling during embryogenesis.
Medium		*LTBP2*	*VRTN*	0.744	0	Extracellular matrix formation in mammary glands.
Low		*NPHP1*	*AREL1*	0.557	0	Ciliary function and organ development.
Low		*MKX*	*PROX2*	0.502	0	Differentiation of connective tissues and tendons.
Low		*BMP2*	*VRTN*	0.454	0	Bone development related to mammary spacing.
**Litter uniformity trait**	**142**					
High		*SMAD4*	*SMAD2*	0.999	0.084	Signal transduction for uniform embryonic development.
High		*ACVR2B*	*INHBA*	0.984	0.049	Regulation of cell proliferation and fetal growth.
High		*APOB*	*APOE*	0.988	0.272	Lipid transport and placental nutrition to the fetuses.
Medium		*BMP2*	*SMAD4*	0.845	0	Tissue differentiation and body axis formation.
Medium		*IGF1*	*BMP2*	0.791	0.071	Key growth factor influencing birth weight.
Medium		*ARAP2*	*TSN*	0.778	0	Cytoskeletal remodeling during development.
Low		*IL1B2*	*TNFAIP3*	0.578	0.174	Regulation of inflammation and maintenance of a healthy uterine environment.
Low		*NSRP1*	*DOCK7*	0.453	0	Cell division processes and neuronal migration.
Low		*ACVR2B*	*IGF1*	0.427	0	Indirect interaction within systemic growth pathways.
**Litter size trait**	**92**					
High		*ACVR2A*	*INHBA*	0.987	0.077	Regulation of FSH and ovulation rate.
High		*ESR1*	*ESR2*	0.974	0	Estrogen receptors essential for uterine maintenance.
High		*ACVR1*	*BMPR1B*	0.962	0	*BMPR1B* is a major gene known to increase fecundity.
Medium		*IGF1R*	*ESR1*	0.914	0.084	Intersection between nutrition and fertility (somatotropic axis).
Medium		*BMP7*	*BMPR1B*	0.853	0	Follicular development and oocyte maturation.

Medium		*ESR1*	*KISS1R*	0.756	0	Neuroendocrine control of estrous cycle initiation.
Low		*TGFBR2*	*ZFYVE9*	0.702	0	Mediator of TGF-β signaling during embryogenesis.
Low		*MFN1*	*TFAM*	0.685	0	Mitochondrial function required for oocyte viability.
Low		*BMPR1B*	*TGFBR2*	0.497	0.147	Interaction in reproductive tissue remodeling.
**Carcass and Meat quality trait**	**196**					
High		*ACACA*	*FASN*	0.999	0.207	Key enzymes for de novo fatty acid synthesis.
High		*ACACA*	*PRKAG3*	0.908	0	*PRKAG3* regulates glycogen content and meat pH (Napole effect).
High		*SCD*	*FASN*	0.941	0.354	Determines the composition of saturated and unsaturated fats.
Medium		*SREBF1*	*SCD*	0.871	0.053	Master transcription factor of lipogenesis.
Medium		*FABP1*	*ACACA*	0.459	0	Fatty acid transport into the cell.
Medium		*UGGT1*	*PRPF40A*	0.697	0.072	Protein quality control and cellular stress in muscle.
Low		*ADIPOQ*	*VCAM1*	0.445	0.169	Relationship between adiponectin and adipose tissue inflammation.
Low		*VDR*	*SREBF1*	0.428	0.057	The vitamin D receptor modulates fat accumulation.
Low		*SQLE*	*ACACA*	0.406	0.06	Cholesterol biosynthesis pathway and lipid precursors.
